# Accuracy and correlates of maternal recall of birthweight and gestational age

**DOI:** 10.1111/j.1471-0528.2008.01717.x

**Published:** 2008-06

**Authors:** ARA Adegboye, BL Heitmann

**Affiliations:** a University of Southern Denmark, Institute of Sports Science and Clinical Biomechanics, Research in Childhood HealthDenmark; b Copenhagen University Hospital, Institute of Preventive Medicine, Research Unit for Dietary StudiesDenmark

**Keywords:** Birthweight, data linkage, gestational age, maternal recall, validation

## Abstract

**Objective:**

To determine the accuracy of maternal recall of children birthweight (BW) and gestational age (GA), using the Danish Medical Birth Register (DBR) as reference and to examine the reliability of recalled BW and its potential correlates.

**Design:**

Comparison of data from the DBR and the European Youth Heart Study (EYHS).

**Setting:**

Schools in Odense, Denmark.

**Population:**

A total of 1271 and 678 mothers of school children participated with information in the accuracy studies of BW and GA, respectively. The reliability sample of BW was composed of 359 women.

**Method:**

The agreement between the two sources was evaluated by mean differences (MD), intraclass correlation coefficient (ICC) and Bland–Altman's plots. The misclassification of the various BW and GA categories were also estimated.

**Main outcome measures:**

Differences between recalled and registered BW and GA.

**Results:**

There was high agreement between recalled and registered BW (MD =−0.2 g; ICC = 0.94) and GA (MD = 0.3 weeks; ICC = 0.76). Only 1.6% of BW would have been misclassified into low, normal or high BW and 16.5% of GA would have been misclassified into preterm, term or post-term based on maternal recall. The logistic regression revealed that the most important variables in the discordance between recalled and registered BW were ethnicity and parity. Maternal recall of BW was highly reliable (MD =−5.5 g; ICC = 0.93), and reliability remained high across subgroups.

**Conclusion:**

Maternal recall of BW and GA seems to be sufficiently accurate for clinical and epidemiological use.

*Please cite this paper as:* Adegboye A, Heitmann B. Accuracy and correlates of maternal recall of birthweight and gestational age. BJOG 2008;115:886–893.

## Introduction

Birthweight (BW) and gestational age (GA) are recognised as important measures of pregnancy outcomes.^1^ Evidence is accumulating to show that BW and GA are also associated with health throughout the lifespan, supporting the fetal origins hypothesis of adult diseases.^2^

BW and GA can be measured and registered as part of the routine medical record. However, recorded information may be unavailable if a child was born a number of years ago, at home or in areas where hospital birth records are not obtained or where there are problems with data quality. Additionally, hospital or state records are not available for deliveries occurring outside the country. For these reasons, in epidemiological studies, maternal recall is often the only feasible means by which information can be obtained. Therefore, it is important to assess the accuracy and reliability of maternal recall by comparison with direct measurement.

To date, the accuracy of maternal report of BW^3^–^8^ has been subject of more attention than maternal report of GA.^9^,^10^ Furthermore, previous studies have focused mainly on the accuracy of maternal recall, and only few studies have examined both the accuracy and reliability of recalled BW.^3^,^9^ Epidemiological studies have demonstrated that accuracy of maternal recall differs significantly among populations.^7^ The accuracy of recalled and registered information on BW range markedly from 71% of exact agreement (USA)^11^ to 16% of agreement within BW groups (China).^12^ Thus, it is desirable to validate the responses given in questionnaires among subsamples of target populations.

The aims of this study were to examine the accuracy of maternal recall of BW and GA in a Danish population and to identify their correlates. In addition, reliability of maternal recall of BW was assessed.

## Methods

### Study design

The Danish part of the European Youth Heart Study (EYHS) is a longitudinal study of the associations between lifestyle and risk factors for cardiovascular disease in children, from which boys and girls in the third and ninth grades were recruited in 1997–98 in Odense county, DK. The children at third grade were followed for 6 years (2003/04), when a new third grade cohort was introduced. Complete information on the cohorts is presented elsewhere.^13^,^14^

Mothers of participating children completed a questionnaire on the child's BW in g at both baseline and follow up. Maternal information on child's GA in weeks was collected at follow up only. Parents were also asked about their socio-demographic characteristics, lifestyle and current weight and height.

The study was approved by the local scientific ethics committee. All parents gave written informed consent and all children gave verbal consent.

### Data linkage

Recalled information on BW and GA was compared with registered information in the national Danish Medical Birth Register (MBR). The EYHS was linked with MBR database by matching the national identification number (unique 10-digit identity number). The MBR was established in 1968 and has been computerised since 1973. This register contains information of all births in Denmark and is considered of good quality.^15^

### Study population

A total of 1537 children and parents participated in the EYHS. Of these, 1448 respondents were the biological mother of the participating child and 1428 mothers recalled, at least, once their children's BW and 359 recalled both at baseline and at the 6-year follow up. The first maternal recall on child's BW was preferably used for the accuracy analyses. Maternal recall at follow up was used in the absence of BW information at baseline (32%).

Regarding accuracy analysis of BW, a total of 157 children were excluded due to missing information on BW in the MBR database (incomplete identity number or born outside Denmark), leaving a final accuracy sample of 1271 women. The reliability sample of BW was composed of 359 women.

Of 749 biological mothers who provided information on children's GA, a total of 72 children were excluded due to missing information in the MBR database. Therefore, 678 women constituted the accuracy sample for GA.

### Statistical analysis

Women who did not remember their children's BW were compared with the remaining cohort of women with, at least, one recalled BW using chi-square test and analysis of variance. The same procedure was applied for those who did not recall GA. However, the analysis was restricted to follow up data because maternal information on children's GA was collected at follow up only.

The discrepancy between recalled and registered BW and GA was assessed by Student's *t* test (mean difference [MD]; SD). Positive values represent overestimation and negative values represent underestimation of true value. Student's *t* test was also used for testing the difference between second recall and first maternal recall of BW.

Correlation and agreement between recalled and registered information on BW and GA and second versus first maternal recall on BW for the overall sample and across groups were investigated using Pearson's coefficient correlation (*r*) and intraclass correlation coefficient (ICC).

Recalled and registered BWs and GAs were also compared using Bland–Altman plot,^16^ which consists of a graphical display of the differences (recalled − registered) against their MD (recalled + registered/2), 95% limits of agreement and respective confidence intervals. The 95% limits of agreement (MD ± 1.96 SD of the differences) identify the range of scores in which 95% of the differences between the two measurement methods are expected to fall. Moreover, the Bland–Altman plot was used to identify causes of discrepancies (observations outside the limits of agreement) between maternally recalled and registered information.

Linear regression analysis was used to assess the relationship between registered (dependent variable) and recalled BW and GA (independent variable). We tested the hypothesis that the coefficients β_0_ and β_1_ correspond to 0 and 1, respectively, which indicates a perfect fit. Stepwise multiple logistic regressions were used to relate the odds of being outside the limits of agreement and of having a discrepancy of more than 100 g in BW and 2 weeks in GA. Cutoffs of 100 g and 2 weeks were chosen because they represent differences in BW and GA of physiological significances.^7^

Also, sensitivity of maternal recall for detecting BW and GA groups, classified according to cutoffs of clinical significance, was presented: low (<2500 g), normal (2500–4000 g) and high BW (>4000 g) and preterm (<37 weeks), term (37–41 weeks) and post-term (≥42 weeks). Based on age- and-sex-adjusted weight categories from a Danish reference population,^17^ we classified infants, who were born between 27 and 43 weeks of gestation, as being small for gestational age (SGA) (≤10th percentile), adequate for gestational age (AGA) or large for gestational age (LGA) (≥ 90th percentile). Kappa coefficients were applied to evaluate the magnitude of agreement, for example kappa values of 0.8 and greater represented ‘excellent’ agreement, 0.61–0.8 ‘substantial’ and 0.41–0.6 ‘moderate’ agreement.^18^ Moreover, we evaluated the quality of BW and GA information on the MBR. The percentage of implausible values for GA and BW-GA combinations was calculated according to cutoffs proposed by Alexander *et al*.^19^,^20^ The analyses were performed using Stata 9.0 (StataCrop, TX, USA).

## Results

Except for current body mass index (BMI), no differences were found for socio-demographic and birth-related characteristics between the women who recalled and those who did not recall children's BW, for example nonresponders had higher BMI than responders. Regarding missing recall on GA, significant differences were detected in relation to the child's age, ethnicity, maternal age, education and parity. Child's age and maternal age and parity were higher in the nonrespondent group. The proportion of nonwhite children and low-educated women were also higher among nonparticipants than among participants (Table 1).

**Table 1:** Comparison of maternal and children's characteristics between biological mothers who recalled and did not recall birthweight or gestational age

Characteristics	Birthweight*			Gestational age**		
		
	Respondents (*n* = 1428)	Nonrespondents (*n* = 20)	*P* value	Respondents (*n* = 748)	Nonrespondents (*n* = 98)	*P* value
**Child's age**						
8–11 years	68%	62%	0.5	94%	87.8%	0.02
14–18 years	32%	38%		6.0%	12.2%	
**Child's gender**						
Boy	46%	47%	0.9	44%	42%	0.75
Girl	54%	53%		56%	58%	
**Child's ethnicity**						
White	94%	83%	0.07	96%	77%	0.0001
Nonwhite	6%	17%		4%	23%	
**Maternal education**						
Less than college	65%	47%	0.1	60%	77%	0.001
College or more	35%	53%		40%	23%	
**Maternal civil status**						
Married	72%	80%	0.6	74%	80%	0.4
Single	28%	20%		26%	21%	
**Maternal age**	40.4 (5.3)	40.6 (3.7)	0.8	39.2 (4.6)	40.2 (5.1)	0.04
**Parity**	1.7 (0.8)	1.9 (1.0)	0.4	1.6 (0.8)	1.9 (0.8)	0.02
**Maternal current BMI**	23.8 (4.1)	26.6 (12.1)	0.006	23.8 (4.1)	24.1 (6.0)	0.5

Analyses are restricted to biological mothers. Analysis of variance (mean [SD]) and chi-square test were performed.

* Comparison between women who did not recall and who recalled at least once their children's BWs (EYHS; *n* = 1448).

** Comparison between women who did not recall and who recalled their children gestational age at birth. Analyses restrict those who participated at follow up (EYHS; *n* = 846).

BW, as recorded by MBR, ranged from 866 to 5200 g (MD 3388 g, SD 567.1), with some evidences that the figures had been rounded off to 0. GA ranged from 26 weeks to 45 weeks and (MD 39.6 weeks, SD 1.9). No implausible values for GA (<20 weeks or >50 weeks)^19^ and BW–GA combinations^20^ were found.

### Correlations and discrepancies across groups

In total, 68 and 42%, respectively, of the maternally recalled BWs and GAs were completely identical to those recorded on the MBR. Additionally, 92% of the BWs were recalled within 100 g of the registered BWs, and 94% of the GAs were recalled within 2 weeks of registered GAs (Table 2).

**Table 2:** Distribution of the difference between maternally recalled BW and GA and recorded information on the MBR

Absolute difference	EYHS	
	
	Frequency	Percent
**BW, g (*n* = 1271)**		
0	861	68
50	1074	84
100	1166	92
**GA, weeks (*n* = 678)**		
0	284	42
1	585	86
2	640	94

Overall, there was a slight tendency for women to underestimate their children BWs (MD −0.2 g, SD 142.4) and overestimate GA (MD 0.3 weeks, SD 1.9). No significant difference in mean discrepancies between maternal recall and registered BWs across subgroups were detected. A significant underestimation of GA was detected among mothers of nonwhite children and single mothers. Mothers who gave birth to SGA (MD −0.1 weeks, SD 1.5) babies underestimated the GA compared with those who gave birth to AGA (MD 0.3 weeks, SD 1.1; *P* = 0.048) and LGA babies (MD 0.4 weeks, SD 1.2; *P* = 0.006). A significant difference was detected between maternal BMI and maternal educational groups. Overweight and higher educated mothers significantly overestimated the GA compared with others.

The overall Pearson's correlation coefficients and ICC on BW were 0.97 and 0.94, respectively, and varied little only across subgroups. The overall correlation coefficients and ICC on GA were 0.85 and 0.76, respectively, and were markedly lower (*r* = 0.37; ICC = 0.42) in the group of children who were born after 41 weeks (Table 3).

**Table 3:** Correlation, agreement and mean discrepancy (MD) between recalled and registered BW and GA in a sample from EYHS

Characteristics	EYHS cohort					
	
	BW (*n* = 1271)			GA (*n* = 678)		
		
	r	ICC	MD (SD)	r	ICC	MD (SD)
**Overall**	0.97	0.94	−0.2 (142.4)	0.85	0.76	0.3 (1.9)
**Child's age**						
8–11 years	0.98	0.95	1.2 (127.7)	0.85	0.76	0.3 (1.2)
14–18 years	0.95	0.88	−3.9 (174.8)	—	—	—
**Child's gender**						
Boy	0.96	0.97	5.8 (179)	0.85	0.76	0.3 (1.2)
Girl	0.98	0.97	−5.5 (100.1)	0.85	0.76	0.2 (1.2)
**Child's ethnicity**						
White	0.97	0.94	−0.4 (136.5)	0.86	0.77	0.3 (1.2)*
Nonwhite	0.82	0.63	10.8 (256.2)	0.76	0.64	−0.7 (1.7)
**BW groups**						
Low	0.91	0.69	27.9 (207.0)	0.93	0.86	−0.07 (1.2)
Normal	0.93	0.86	−3.2 (144.8)	0.71	0.56	0.3 (1.2)
High	0.97	0.94	8.0 (55.0)	0.80	0.72	0.4 (0.9)
**GA groups**						
Preterm	0.99	0.99	3.2 (86.8)	0.88	0.78	0.1 (1.4)
Term	0.96	0.92	−3.0 (136.6)	0.64	0.48	0.3 (1.2)
Post-term	0.97	0.92	15.7 (128.2)	0.37	0.42	0.0 (1.0)
**BW-for-GA**						
SGA	0.99	0.98	0.6 (59.8)	0.88	0.78	−0.1 (1.5)*
AGA	0.97	0.95	−0.6 (98.7)	0.82	0.72	0.3 (1.1)
LGA	0.92	0.82	7.7 (186.9)	0.75	0.65	0.4 (1.2)
**Maternal age**						
20–39 years	0.97	0.95	2.9 (121.3)	0.81	0.70	0.3 (1.2)
≥40 years	0.97	0.94	−2.1 (158.7)	0.89	0.82	0.2 (1.2)
**Maternal education**						
Less than college	0.96	0.93	4.8 (156.1)	0.84	0.75	0.2 (1.3)*
College or more	0.99	0.98	−6.7 (89.1)	0.86	0.80	0.4 (1.0)
**Maternal civil status**						
Married	0.97	0.95	0.7 (134.7)	0.90	0.85	0.2 (1.0)*
Single	0.93	0.85	9.5 (208.6)	0.66	0.51	−0.1 (1.7)
**Previous child**						
Yes	0.96	0.92	0.18 (171.8)	0.82	0.72	0.2 (1.2)*
No	0.98	0.96	−0.6 (104.7)	0.87	0.80	0.3 (1.1)
**Maternal BMI**						
Underweight	0.99	0.99	7.3 (18.1)	0.79	0.62	−0.2 (1.2)*
Normal weight	0.98	0.96	−4.1 (120.2)	0.85	0.77	0.3 (1.1)
Overweight	0.92	0.87	16.9 (201.3)	0.82	0.72	0.4 (1.1)
Obese	0.98	0.95	−8.1 (119.6)	0.80	0.70	−0.1 (1.7)

MD, mean discrepancy (recalled – registered information); *r*, Pearson's coefficient correlation; –, no estimation due to few observations.

* Statistically significant (*P* < 0.05).

Table 4 shows that among the 359 women who informed about their children's BWs twice (6 years apart), maternal recall was highly reliable (*r* = 0.97; ICC = 0.93; MD =−5.5 g, SD 132.4). Reliability of recall remained high when considered separately by subgroups.

**Table 4:** Reliability of maternal recall of birthweight in a sample from EYHS

Characteristics	Reliability (*n* = 359)		
	
	r	ICC	MD (SD)
**Overall**	0.97	0.93	−5.5 (132.4)
Child's age			
8–11 years	0.97	0.93	−5.5 (132.4)
14–18 years	—	—	—
**Child's gender**			
Boy	0.95	0.87	−9.3 (179.9)
Girl	0.99	0.99	−2.4 (74.3)
**Child's ethnicity**			
White	0.97	0.93	−4.7 (130.3)
Nonwhite	0.91	—	−42.8 (224.4)
**BW groups**			
Low	0.99	0.99	−13.6 (57.3)
Normal	0.94	0.87	−3.3 (138.6)
High	0.99	0.99	0.4 (31.3)
**GA groups**			
Preterm	0.99	—	−5.3 (37.0)
Term	0.96	0.90	−3.2 (139.7)
Post-term	0.99	0.99	−0.27 (36.2)
**BW-for-GA**			
SGA	0.98	0.99	29.7 (101.3)
AGA	0.98	0.97	−0.5 (69.3)
LGA	0.83	0.95	−21.7 (247.5)
**Maternal age**			
20–39 years	0.96	0.89	−11.4 (147.8)
≥40 years	0.99	0.97	8.2 (99.0)
**Maternal education**			
Less than college	0.96	0.91	−3.0 (152.3)
College or more	0.99	0.98	−11.3 (67.3)
**Maternal civil status**			
Married	0.99	0.97	−3.0 (85.6)
Single	0.92	0.80	−12.8 (219.4)
**Previous child**			
Yes	0.99	0.98	5.7 (84.5)
No	0.94	0.83	−12.7 (164.5)
**Maternal BMI**			
Underweight	1.0	—	0 (0.0)
Normal weight	0.96	0.90	−8.9 (154.1)
Overweight	0.99	0.97	−1.2 (68.3)
Obese	0.99	0.97	17.9 (77.9)

MD, mean discrepancy (second recall – first recall); *r*, Pearson's coefficient correlation. —, no estimation due to few observations.

All comparisons were non-statistically significant (*P* > 0.05).

### Linear regression analyses and graphic display of agreement

In the linear regression analyses using registered BW and GA as dependent variables and maternal recall as independent variable, the linear and angular coefficients were significant (*P* = 0.0001)_._ Hence, the hypothesis of perfect fit was rejected, which suggests that maternally recalled BW and GA should be adjusted through linear regression equation.

**Figure 1:**
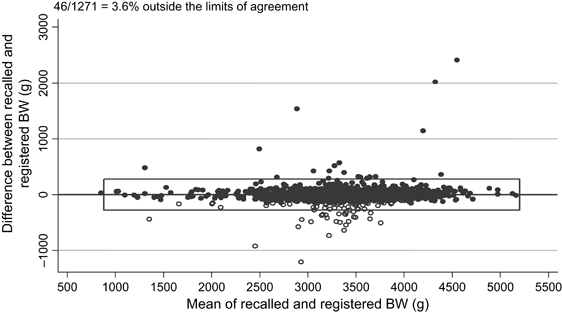
Agreement between recalled and registered BW, with 95% limits of agreement, confidence intervals and regression line.

**Figure 2:**
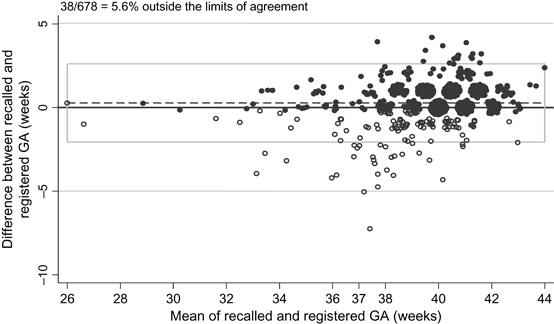
Agreement between recalled and registered GA, with 95% limits of agreement, confidence intervals and regression line.

The Bland–Altman plots for BWs and GAs are given in Figures 1 and 2, respectively. Figure 1 shows that there were small differences only between recalled and registered BW, considering that the majority of the points were located close to the horizontal line, which represents 0. Only 3.6% of the differences were outside the limits of agreement (−285/284.5 g). In comparing the concentration of points above and below 0 (perfect agreement), a slight tendency towards underestimation among normal BW children, especially from 2900 to 3600 g (around the mean value of 3400 g), was observed. Figure 2 also shows that most of the differences between maternally recalled and registered GA aggregated within 95% limits of agreement (5.6% outside the limits; limit bands are −2.1/2.6 weeks). However, it was possible to identify a distinct pattern of agreement according to GA at birth, for example a trend towards underestimation between 36 and 39 weeks of gestation, and a slight trend towards overestimation for post-term infants.

### Explanations for discrepancies

The stepwise analysis including child age, ethnicity, gender, BW, GA, BW-for-GA groups, maternal age, education, civil status, parity and BMI showed that the only factors related to BW differences outside the 95% limits of agreement were having a nonwhite child and having a previous birth. The same variables were associated with having a discrepancy of more than 100 g. A variable indicating whether BW recall was collected at baseline or follow up was also introduced in the models, but no significant effect was observed.

The variables significantly associated with discrepancies in GA were maternal civil status and maternal BMI. Single mothers had a higher likelihood of discrepant recall of GA than married mothers and the likelihood of discrepant recall rose with increasing maternal BMI (Table 5).

**Table 5:** Results of logistic regression showing odds ratios associated with discrepancies between recalled and registered BW and GA

Variables	OR	95% CI	P	OR	95% CI	P
		
	Outside limits			>100 g difference		
**BW**						
Nonwhite child	9.1	2.7–30.1	0.0001	6.1	2.3–16.4	0.0001
Mother with previous child	1.1	1.01–1.2	0.029	2.9	1.6–4.9	0.0001

	Outside limits			>2 weeks difference		
**GA**						
Single mother	10.6	3.2–35.7	0.0001	10.6	3.2–35.7	0.0001
Maternal current BMI	1.2	1.01–1.3	0.032	1.2	1.01–1.3	0.032

Dependent variables: being outside the 95% limits of agreement or having an absolute discrepancy >100 g in birthweight and 2 weeks in gestational age. OR, adjusted odds ratios.

### Sensitivity for detecting BW and GA groups

Examining how errors in maternal recall would affect the classification of children into low-, normal- and high-BW groups show that only 1.6% (20/1271) of births would have been misclassified (kappa 95%; *P* = 0.000). The misclassifications of the GA groups into preterm, term and post-term delivery (17%) and BW-for GA into SGA, AGA and LGA (21%) were higher than misclassification of the BW groups. However, the magnitude of agreement for both GA and BW-for GA groups was moderate (kappa 56 and 58%, respectively, *P* = 0.000) (Table 6).

**Table 6:** The proportion of birthweight and gestational age groups misclassification

Maternal recall	Information from the Danish Birth Register				
	
	Low (*n* = 72)	Normal (*n* = 1061)	High (*n* = 138)	Kappa	Agreement
Low birthweight	94% (68)	0.3% (3)	0	95%*	98%
Normal birthweight	6% (4)	98.7% (1048)	2% (3)		
High birthweight	0	1% (10)	98% (135)		

	Preterm (*n* = 41)	Term (*n* = 571)	Post-term (*n* = 66)		
Preterm delivery	93% (38)	4% (22)	0	56%*	83%
Term delivery	7% (3)	83% (472)	15% (10)		
Post-term delivery	0	13% (77)	85% (56)		

	SGA (*n* = 63)	AGA (*n* = 432)	LGA (*n* = 174)		
SGA	78% (49)	6.7% (29)	2% (3)	58%*	79%
AGA	17% (11)	85% (367)	34% (59)		
LGA	5.0% (3)	8% (36)	64% (112)		

Numbers are given in parentheses.

* Statistically significant (*P* < 0.05).

## Discussion

In this study, we demonstrate a high degree of accuracy and reliability of maternal recall of their children's BWs. Maternally recalled GA was also accurate and the degree of accuracy varied according to the child's ethnicity, BW-for GA groups, maternal civil status, BMI and education. Unexpectedly, mothers with a higher education overestimated the GA compared with less-educated mothers. Although the mean differences were statistically significant, the discrepancies between recalled and registered information were less than 1 week in all groups, which appears to be of little clinical relevance.

Although the overall underestimation of BW (−0.2 g) and overestimation of GA (0.3 weeks) were very small and had low impacts on BW and GA classification groups, it is important to consider that for evaluation of fetal growth combinations of both information resulted in an error of larger magnitude (21%).

The results demonstrate that the magnitude of agreement were higher for BW than for GA. This is not a surprising finding because child BW is the sort of information always awaited by the parents after delivery, and it is often repeated to family and friends, and therefore more likely memorised. GA is not often mentioned after delivery, especially if the child was born at term. Moreover, estimation of GA is more complex and accuracy varies according to the method used.^21^

Although the linear regression analyses suggested that maternal recall of BW and GA should be adjusted through linear regression equation, the models showed a high correlation and a good explanatory capacity. Recalled BW and GA explained 94 and 72% of the variance in registered information, respectively. Whether correction for measurement error would be appropriate on the basis of this validation study is still debatable. If a gold standard measured with error is used to correct another imperfect measurement, this can introduce new bias.^22^ The MBR was used as a standard source of information to validate the maternal recalls. The MBR is not a perfect gold standard; however, it seems to have a good quality.^15^

The logistic regression analysis proved that the most important variable in the discordance between recall-based and register-based information on BW were child's ethnicity and having a previous child. These findings are in accordance with other studies, also showing that multiparous mothers may confuse their children's BWs and that mothers of nonwhite infants recall their children's BW less accurately than mothers of white infants.^7^,^23^ Relevant variables predicting inaccuracy of GA in the logistic models were maternal civil status and BMI. Maternal civil status might be considered as proxy of socio-economic class and family support and maternal BMI as proxy of lifestyle factors and health concern.

Although several methods have been proposed to validate maternal recall of BW and GA information,^3^–^5^,^10^ validation studies are often analysed inappropriately, notably by using correlation coefficient that may be misleading. Pearson's correlation coefficients measure the strength of a correlation or linear relatedness between two variables, but not agreement. Consequently, Pearson's correlation coefficients can reach high values when there is disagreement between two measurements, if the bias is systematic.^16^ This problem can be overcome by using ICC, which combines a measure of correlation with a test in the difference of means (within and between subjects). In the present validation study, the use of various methods simultaneously in the analyses allowed a better view of the importance and the sensitivity grade of each one.

Combining the graphical approach of Bland–Altman with ICC allows for identification of heterogeneous patterns of agreement. A heterogeneous pattern of accuracy through different levels of the registered information can be more easily identified by a quick look at the graph. The presence of heterogeneity indicates the need to estimate ICC for different level of the variable studied (e.g. trend towards underestimation in BW between 2900 and 3600 g). The two methods may complement each other in pilot studies aiming to evaluate the agreement between recalled and registered BW and GA. Identifying groups with reliable information for those variables may justify the use of self-reported values, thus making fieldwork cheaper and easier.

## Conclusion

Although the quality of maternal recall on BW and GA might have a slight importance for clinical practice, it is a relevant issue to future epidemiological research, which may lead to clinically useful information.

The small magnitude of means of the difference between recall-based and register-based information and the low rate of misclassification into BW and GA groups suggest that maternal recall of BW and GA can provide accurate information for epidemiological studies regarding fetal and infant growth.
